# Swallowing trajectories and influencing factors among subacute stroke patients with dysphagia in rehabilitation wards: a longitudinal study

**DOI:** 10.3389/fneur.2026.1916813

**Published:** 2026-09-15

**Authors:** Yanan Zhu, Xiuru Tang, Meng Guo, Min Li, Shiyan Sun, Minmin Zhou, Xiurong Yu

**Affiliations:** 1 Department of Rehabilitation Medicine, Shandong Medical and Pharmaceutical University Hospital, Binzhou, China; 2 School of Nursing, Shandong Medical and Pharmaceutical University, Binzhou, China; 3 Hospital Public Relations Department, Shandong Medical and Pharmaceutical University Hospital, Binzhou, China

**Keywords:** stroke, dysphagia, swallowing rehabilitation, trajectory, influence factor

## Abstract

**Background:**

Rehabilitation training is crucial for subacute stroke patients with dysphagia. However, research on trajectories of clinical swallowing performance and associated influencing factors following admission to rehabilitation wards remains quite limited. This study aimed to identify these trajectories and influencing factors among this specific patient population.

**Methods:**

A total of 143 subacute stroke patients with dysphagia were recruited from the rehabilitation ward between April 2024 and July 2025 by convenience sampling. All patients completed baseline assessments within 24 h of admission using the Standardized Swallowing Assessment (SSA), Oral Health Assessment Tool (OHAT), Nutritional Risk Screening 2002 (NRS 2002), Fatigue Severity Scale (FSS), and Patient Health Questionnaire-9 (PHQ-9). Clinical swallowing performance was then reassessed using the SSA at 7, 14, 21, and 28 days after admission. Latent class growth modeling (LCGM) based on repeated SSA scores over the 4-week follow-up was used to identify distinct clinical swallowing performance trajectories, and multinomial logistic regression was used to analyze their influencing factors.

**Results:**

Based on the longitudinal SSA data, the clinical swallowing performance trajectories of subacute stroke patients with dysphagia in inpatient rehabilitation were classified into three groups: a mild-rapid improvement group (31%), a moderate-steady improvement group (41%), and a severe-slow improvement group (28%). Age, oral health status, depression, and fatigue were significantly associated with trajectory membership.

**Conclusion:**

Subacute inpatients with post-stroke dysphagia exhibit three distinct clinical swallowing performance trajectories, demonstrating significant group heterogeneity. These trajectories may assist in early risk stratification and individualized care planning.

## 1 Introduction

Dysphagia has been classified by the World Health Organization (WHO) as an independent health condition in the International Classification of Diseases, 11th Revision (ICD-11) (1). It is a common and serious complication among stroke patients. A systematic review (2) indicated that the pooled prevalence of post-stroke dysphagia (PSD) was 46.6% globally, with a prevalence of 40.1% in Asia. An epidemiological report (3) from China further indicated that the prevalence of dysphagia was 46.3% during the acute phase of stroke and 56.9% during the recovery phase. Compared with stroke patients without dysphagia, those with dysphagia experience prolonged hospitalization (by 4.73 days) and incur 40.36% higher medical costs (4). They face 4.08-times and 4.07-times increased risks of pneumonia and mortality, respectively (5). In-hospital mortality was more than doubled (6). These complications place a substantial burden on individuals, families, and the healthcare system.

Recovery of swallowing function after stroke relies on neuroplasticity mechanisms (7). After stroke, the nervous system adapts to altered functional demands through synaptic plasticity-mediated circuit reorganization (8). Repeated, sustained, and sufficiently intense sensorimotor stimulation activates cortical and subcortical swallowing-related networks, strengthening synaptic connectivity and promoting functional swallowing recovery (7). In inpatient rehabilitation settings, such training is delivered to stroke patients regularly and in a standardized manner. Compared with the acute phase, which focuses primarily on life support, the subacute phase (7 days to 6 months post-stroke) is characterized by stabilized vital signs and the patient's ability to undergo regular, standardized inpatient rehabilitation of moderate intensity, representing a critical period for systematic swallowing recovery (9). During this critical period, longitudinal assessment of swallowing function is essential for informing rehabilitation planning. However, repeated flexible endoscopic evaluations of swallowing (FEES) and videofluoroscopic swallowing studies (VFSS) are often constrained by specialized operator requirements and high costs. The SSA functions as a bedside screening instrument that evaluates clinical swallowing performance through directly observable indicators. As such, it offers a feasible and repeatable proxy for tracking changes in clinical swallowing performance.

Prior studies suggest that among subacute stroke patients with dysphagia, clinical swallowing performance outcomes vary even when receiving identical standardized rehabilitation, and this variability is influenced by multiple factors (10, 11). Therefore, identifying these factors and understanding their impact on clinical swallowing performance is potentially important for distinguishing different improvement patterns and informing personalized care plans. However, the dynamic process of clinical swallowing performance remains incompletely understood. Existing assessments are largely confined to one or two time points, thus preventing the observation of continuous dynamic changes (12, 13). Moreover, current analytical approaches have relied predominantly on variable-centered methods that assume population homogeneity, potentially overlooking inter-individual heterogeneity in the swallowing improvement process. One prior study has applied person-centered methods to identify distinct clinical swallowing performance trajectories in this population; however, these trajectories span both inpatient and outpatient periods, failing to capture the specific characteristics unique to this inpatient rehabilitation setting (14).

To address these gaps, this study employs a prospective longitudinal design with repeated assessments over a 4-week inpatient rehabilitation period. Participants are subacute stroke patients with dysphagia admitted to rehabilitation wards. We apply latent class growth modeling (LCGM) to identify distinct subgroups of clinical swallowing performance trajectories. Subsequently, logistic regression is used to examine factors associated with trajectory membership. We hypothesize that this population comprises distinct trajectory subgroups, and that factors such as age, oral health status, and psychological status are associated with different subgroup memberships. If externally validated, these findings may assist in early identification of patients at risk for poorer clinical swallowing performance trajectories and could inform individualized care planning. This study characterizes patterns of change in clinical swallowing performance during inpatient rehabilitation, offering a preliminary framework for future risk stratification and care planning.

## 2 Methods

### 2.1 Participants

This study adopted a prospective longitudinal design. Participants were recruited using convenience sampling from subacute stroke patients with dysphagia admitted to the Rehabilitation Medicine Department at Shandong Medical and Pharmaceutical University Hospital, China, between April 2024 and July 2025. During the recruitment period, research staff consecutively screened all patients admitted to the rehabilitation unit against predefined inclusion and exclusion criteria. Eligible patients and their family members were promptly contacted upon identification. All patients who met the inclusion criteria were invited to participate, and no additional selection procedures were applied. Prior to obtaining informed consent, research staff provided participants with comprehensive information regarding the study objectives, procedures, anticipated benefits, and potential risks, and explicitly stated that participants retained the right to withdraw at any time without affecting their routine clinical care. Inclusion criteria were: (a) meeting the diagnostic criteria for stroke (15), confirmed by cranial CT or MRI imaging; (b) a Water Swallowing Test score ≥ grade 3, and dysphagia confirmed by Flexible Endoscopic Evaluation of Swallowing (FEES); (c) disease duration between 7 days and 6 months; (d) stable vital signs and essentially intact cognitive function; and (e) written informed consent provided by both the patient and their family members. Exclusion criteria were as follows: (a) pre-existing dysphagia before stroke onset; (b) presence of severe organ dysfunction such as heart, liver, kidney, or other vital organs; and (c) severe hearing impairment or sensory aphasia.

Sample size estimation was performed using G^*^Power 3.1.9.7 (Franz Faul, Universität Kiel, Kiel, Germany), based on repeated-measures analysis of variance (ANOVA). Sample size estimation was performed using G^*^Power 3, based on repeated-measures analysis of variance (ANOVA). This approach is grounded in the power analysis principles of *F*-tests under longitudinal repeated-measures designs. With a prespecified type I error rate, statistical power, expected effect size, number of repeated measurements, and within-subject correlation, the required sample size was derived using the non-central *F* distribution. The following parameters were specified: effect size *f* = 0.3 (medium effect), significance level α = 0.05 (two-tailed), statistical power (1–β) = 0.80, and five measurement time points. The analysis yielded a minimum sample size of 134. To account for an anticipated attrition rate of 15%, the target sample size was set to 158. During the recruitment period, 169 individuals were screened for eligibility, of whom 10 did not meet the inclusion criteria. After excluding these 10 ineligible individuals, 159 participants were enrolled and completed the baseline assessment.

### 2.2 Rehabilitation protocol

All participants received a standardized swallowing rehabilitation program once daily. Restorative exercises comprised: (1) Oral ice stimulation (20 min), in which a speech-language pathologist applied an ice-coated cotton swab sequentially to the soft palate, tongue base, and posterior pharyngeal wall; (3) neuromuscular electrical stimulation (20 min) applied to the suprahyoid muscles by a speech-language pathologist; (3) finger acupressure (20 min) performed by a trained rehabilitation therapist at nine acupoints (Jiache, Yingxiang, Renzhong, Chengjiang, Futu, Lianquan, Shanglianquan, Kouheliao, Renying, and Dicang), using intermittent pressure (30–60 s per point) to elicit localized soreness (deqi); (4) orofacial muscle training guided by a speech-language pathologist, including lip exercises and tongue exercise. Compensatory strategies consisted of postural swallowing maneuvers instructed by a speech-language pathologist, including chin-down, chin-up, and head rotation (left and right) during dry swallow. All interventions were tailored to patient tolerance and swallowing function.

### 2.3 Assessment tools

#### 2.3.1 General information questionnaire

The General Information Questionnaire was developed by the research team based on a literature review and group discussion. It comprised two sections: demographic information and disease-related information. Demographic information included age, sex, marital status, smoking history, and place of residence. Disease-related information included stroke type, lesion side, disease duration, the presence of diabetes, hypertension, and dysarthria. Because this questionnaire collected only objective information, it did not undergo formal psychometric validation.

#### 2.3.2 Standard swallowing assessment (SSA)

Clinical swallowing performance was assessed using the SSA (16). This scale comprises three components: clinical examination, the 5 mL water swallowing test, and the 60 mL water swallowing test. Total scores range from 18 to 46, with lower scores indicating better function. The test was immediately terminated if the patient exhibited coughing or wheezing. The Cronbach's α coefficient of the SSA was 0.917.

#### 2.3.3 Oral health assessment tool (OHAT)

The oral health status was assessed by the OHAT. The scale was revised and validated by Chalmers et al. (17), and was translated into Chinese by Wang et al. (18). The scale contains eight items: lips, tongue, gums, saliva, natural teeth, dentures, oral hygiene, and toothache. Each item is scored from zero to two points, with a total score ranging from zero to sixteen. Lower scores indicate better oral health status. The Cronbach's α coefficient of the scale is 0.710 and the test-retest reliability is 0.811.

#### 2.3.4 Fatigue severity scale (FSS)

Fatigue was assessed using the FSS. The FSS was developed by Krupp et al. (19) and subsequently translated into Chinese by Wu and Wang (20). It is a unidimensional scale comprising nine items, each rated on a seven-point Likert scale ranging from one (strongly disagree) to seven (strongly agree). Total possible scores range from nine to sixty-three, with higher scores indicating greater fatigue severity. A score of ≥36 is considered indicative of post-stroke fatigue. The Cronbach's α coefficient of the FSS is 0.929.

#### 2.3.5 Patient health questionnaire-9 (PHQ-9)

The depression symptoms of patients were evaluated using the PHQ-9. This scale was developed by Spitzer et al. (21) and was translated into Chinese by Bian et al. (22). It consists of nine items scored on a four-point Likert scale, with responses ranging from zero (not at all) to three (nearly every day). The total score ranges from zero to twenty-seven, and a score of ≥5 indicates the presence of depressive symptoms. The Cronbach's α coefficient of this scale is 0.850.

#### 2.3.6 Nutritional risk screening 2002 (NRS 2002)

Nutritional risk was assessed using the NRS 2002. The NRS 2002 was developed by the European Society of Parenteral Nutrition (ESPEN) in collaboration with scholars from Denmark and Switzerland (23). It comprises three components: nutritional status, disease severity, and age. The total score ranges from zero to seven, with a score of < 3 indicating normal nutritional status and a score of ≥3 indicating the presence of nutritional risk. The inter-rater reliability of the scale is 0.861, and the test-retest reliability is 0.956.

### 2.4 Data collection and quality control

A research team comprising six members was formed, including one attending physician, one head nurse, three senior nurses, and one graduate student. The head nurse served as the team leader and provided standardized training to all members on data collection protocols and patient information confidentiality. All members passed a competency assessment before data collection began.

We implemented a standardized protocol for data collection. After documentation of informed consent, research personnel retrieved relevant sociodemographic and medical information from the institutional electronic health record system. Baseline assessments (T1) were completed within 24 h, encompassing the SSA, FSS, OHAT, NRS-2002, and PHQ-9. Swallowing function was monitored repeatedly using the SSA on days 7 (T2), 14 (T3), 21(T4), and 28 (T5) post-admission. For patients with impaired communication capacity, proxy responses were obtained from their primary caregivers based on direct observation of daily behaviors and clinical symptoms.

To ensure data reliability, patients and their caregivers reviewed completed questionnaires for accuracy. All assessments were performed independently by two trained researchers; discrepancies were resolved through consensus. Verified data were then entered independently by two researchers into a secure database, with personal identifiers replaced by unique study codes.

### 2.5 Statistical methods

Statistical analysis of the data was performed using IBM SPSS 26.0 (IBM Corp., Armonk, NY, USA). Normality of continuous measures was first examined using the Shapiro–Wilk procedure. Normally distributed were reported as mean ± standard deviation, and among groups differences were evaluated via one-way ANOVA. Non-normally distributed continuous measures were described by median with interquartile range, and across-group comparisons were assessed using the Kruskal–Wallis *H*-test. Categorical outcomes were tabulated as frequencies and proportions, with group differences tested via the chi-square or Fisher's exact test as appropriate. A multinomial logistic regression analysis was performed to identify predictors of swallowing function trajectory classification. Statistical significance was defined as a two-sided *P* value < 0.05.

The LCGM was employed to identify distinct trajectories of swallowing function among stroke patients with dysphagia during hospitalization. A series of nested models was estimated, beginning with a one-class specification and incrementing the class count. Model selection balanced statistical evidence against substantive meaningfulness. We evaluated relative fit using the Akaike Information Criterion (AIC), Bayesian Information Criterion (BIC), and sample-size adjusted BIC (aBIC), for which lower values denote superior model fit. Entropy, scaled from zero to one, served as an indicator of classification uncertainty, with higher values corresponding to more precise assignment. The Lo-Mendell-Rubin adjusted likelihood ratio test (LMRT) and bootstrapped likelihood ratio test (BLRT) assessed whether adding a class significantly improved fit; significance at *P* < 0.05 favored the *k*-class solution over the (*k* – 1) class alternative.

## 3 Results

### 3.1 General information

A total of 159 patients were enrolled in this study. Of these, 143 (90.0%) completed all scheduled assessments, while 16 (10.0%) were lost to follow-up. Attrition was accounted for by clinical deterioration (*n* = 2), early discharge (*n* = 11), and request to withdraw (*n* = 3). The sociodemographic and clinical characteristics of the study participants are presented in Table 1.

**Table 1:** Sociodemographic and clinical characteristics in subacute post-stroke dysphagia patients.

Characteristic	Categories	Total (*n* = 143)
Age (years, $\bar{x}\;\pm \;s$)		63.90 ± 6.650
Disease duration[days, median (IQ)]		28 (19, 40)
Gender	Male	75 (52.4)
	Female	68 (47.6)
Residence	Urban	82 (57.3)
	Rural	61 (42.7)
Education level	Primary school or below	39 (27.3)
	Junior or senior high school	69 (48.3)
	College or above	35 (24.4)
Stroke type	Ischemic stroke	92 (64.3)
	Hemorrhagic stroke	51 (35.7)
Diabetes	No	102 (71.3)
	Yes	41 (28.7)

Continuous data are presented as median (interquartile range) or mean ± standard deviation, while categorical data are presented as *n* (%).

### 3.2 Latent classes and characteristics of clinical swallowing performance trajectories in patients with subacute post-stroke dysphagia

In this study, the Standardized Swallowing Assessment (SSA) scores for 143 patients with subacute post-stroke dysphagia were 32.52 ± 2.41, 31.01 ± 3.07, 29.30 ± 3.50, 26.73 ± 3.48, and 25.72 ± 3.26 at T1 through T5, respectively. These repeated measures were incorporated as observed indicators into the latent class growth model, and models containing one to five classes were fitted stepwise, as detailed in Table 2. Model selection results indicated that the three-class model demonstrated lower AIC, BIC, and aBIC values compared with models containing one and two classes, and the LMRT supported the three-class model over the two-class model (*P* < 0.05). Additionally, the three-class model yielded an entropy value of 0.978, exceeding the 0.80 threshold, indicating good classification precision. The class probabilities were 28%, 31%, and 41%, respectively, all meeting the minimum criterion of 10%. By contrast, both the four-class and five-class models contained a class with a probability of 10%, suggesting that increasing the number of classes failed to yield substantively meaningful subgroup distinctions. Consequently, the final model comprised three distinct clinical swallowing performance trajectories in patients with subacute post-stroke dysphagia.

**Table 2:** Latent class model fitting results of clinical swallowing performance in subacute post-stroke dysphagia patients.

Class no.	AIC	BIC	aBIC	Entropy	LMRT (*P*)	BLRT (*P*)	Class probability (%)
1	3669.705	3693.408	3668.095	–	–	–	1
2	3100.588	3136.142	3098.172	0.946	0.0007	< 0.001	0.45/0.55
3	2789.697	2837.103	2786.476	0.978	< 0.001	< 0.001	0.28/0.31/0.41
4	2698.284	2757.541	2694.257	0.954	0.0262	< 0.001	0.31/0.38/0.21/0.10
5	2580.835	2651.943	2576.003	0.958	0.0377	< 0.001	0.28/0.17/0.19/0.10/0.26

Trajectories were named based on the initial severity and changing trend of dysphagia, with results shown in Figure 1. The three trajectories were named as follows: the mild-rapid improvement group (C1; *n* = 44, 31%), characterized by the mildest initial dysphagia and the most rapid improvement; the moderate-steady improvement group (C2; *n* = 59, 41%), characterized by moderate initial dysphagia and a steady improvement; and the severe-slow improvement group (C3; *n* = 40, 28%), characterized by the most severe initial dysphagia and the slowest improvement.

**Figure 1:**
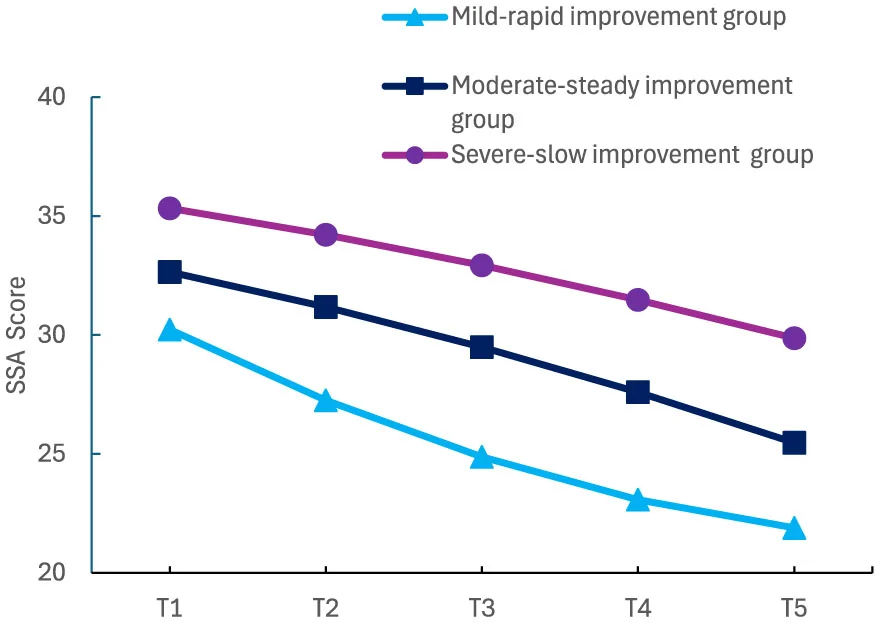
Trajectory of clinical swallowing performance in subacute post-stroke dysphagia patients.

### 3.3 Univariate analysis of clinical swallowing performance trajectories in patients with subacute post-stroke dysphagia

Univariate analysis revealed that age, marital status, smoking history, lesion side, disease duration, hypertension, mRS, NRS2002, dysarthria, fatigue, depression, OHAT, and NIHSS were significantly associated with clinical swallowing performance trajectories (all *P* < 0.05). Detailed results are presented in Table 3.

**Table 3:** Univariate analysis of clinical swallowing performance trajectories in subacute post-stroke dysphagia patients.

Variables	Categories	Mild-rapid improvement group (*n* = 44)	Moderate-steady improvement group (*n* = 59)	Severe-slow improvement group (*n* = 40)	Statistics	*P*
Age (years, $\bar{x}\pm s$)		60.61 ± 6.270	63.90 ± 5.904	67.50 ± 6.353	*F* = 13.158	< 0.001
Gender	Male	17(38.6)	36(61.0)	22(55.0)	χ^2^ = 5.207	0.074
	Female	27(61.4)	23(39.0)	18 (45.0)		
Residence	Rural	18(40.9)	30(50.8)	13 (32.5)	χ^2^ = 3.360	0.186
	Urban	26(59.1)	29(49.2)	27 (67.5)		
Education level	Primary school or below	12(27.3)	15(25.4)	12 (30.0)	χ^2^ = 5.308	0.257
	Junior or senior high school	23(52.3)	24(40.7)	22(55.0)		
	College or above	9(20.4)	20(33.9)	6(15.0)		
Marital status	Married	41(93.2)	53(89.8)	30(75.0)	χ^2^ = 6.119	0.048
	Others (unmarried, divorced, or widowed)	3(6.8)	6(10.2)	10(25.0)		
Smoking history	No	33(75.0)	34(57.6)	19(47.5)	χ^2^ = 6.874	0.032
	Yes	11(25.0)	25(42.4)	21(52.5)		
Stroke type	Ischemic stroke	31(70.5)	37(62.7)	24(60.0)	χ^2^ = 1.113	0.573
	Hemorrhagic stroke	13(29.5)	22(37.3)	16(40.0)		
Lesion side	Unilateral	42(95.5)	47(79.7)	31(77.5)	χ^2^ = 7.032	0.029
	Bilateral	2(4.5)	12(20.3)	9(22.5)		
Disease duration [days, median (IQ)]		24(16, 33)	27(21, 32)	30(22, 57.5)	*H* = 6.927	0.031
Diabetes	No	32(72.7)	43(72.9)	27(67.5)	χ^2^ = 0.398	0.819
	Yes	12(27.3)	16(27.1)	13(32.5)		
Hypertension	No	24(54.5)	26(44.1)	11(27.5)	χ^2^ = 6.372	0.042
	Yes	20(45.5)	33(55.9)	29(72.5)		
mRS	< 3	26(59.1)	24(40.7)	8(20.0)	χ^2^ = 13.281	0.001
	≥3	18(40.9)	35(59.3)	32(80.0)		
NRS2002	< 3	30(68.2)	29(49.2)	17(42.5)	χ^2^ = 6.193	0.045
	≥3	14(31.8)	30(50.8)	23(57.5)		
Dysarthria	No	20(45.5)	23(39.0)	7(17.5)	χ^2^ = 7.914	0.019
	Yes	24(54.5)	36(61.0)	33(82.5)		
Fatigue	No	31(70.5)	20(33.9)	6(15.0)	χ^2^ = 28.368	< 0.001
	Yes	13(29.5)	39(66.1)	34(85.0)		
Depression	No	36(81.8)	32(54.2)	15(37.5)	χ^2^ = 17.495	< 0.001
	Yes	8(18.2)	27(45.8)	25(62.5)		
OHAT [points, median (IQ)]		3(2, 4)	4(3, 6)	6(5, 10)	*H* = 6.927	0.031
NHISS (points, $\bar{x}\pm s$)		8.48 ± 4.433	11.49 ± 5.319	14.43 ± 5.252	*F* = 14.591	< 0.001

Continuous data are presented as median (interquartile range) or mean ± standard deviation, while categorical data are presented as *n* (%).

### 3.4 Multivariable analysis of clinical swallowing performance trajectories in patients with subacute post-stroke dysphagia

A multinomial logistic regression model was used to identify predictors of swallowing recovery trajectories (reference: C1, mild-rapid improvement). Variables with *P* < 0.05 in univariate analysis were entered into the full model. The likelihood ratio test revealed that marital status, smoking history, lesion side, disease duration, hypertension, mRS, NRS2002, dysarthria, and NIHSS were not statistically significant (*P* > 0.05). Moreover, the full model was subject to overfitting due to the large number of independent variables (df = 26) relative to the sample size (*n* = 143), with the smallest group comprising only 40 cases and an EPV of approximately 1.5, far below the recommended threshold of 10 according to the events-per-variable rule. Based on these results, the non-significant variables were excluded, and four core covariates with significant independent contributions (age, OHAT score, depression, and fatigue) were retained for the final parsimonious model. The overall likelihood ratio test revealed that the final model was statistically significant (χ^2^ = 93.608, df = 8, *P* < 0.001), and the Nagelkerke pseudo-*R*^2^ was 0.542, indicating acceptable explanatory capacity of the model. The results of the final multivariable model are presented in Table 4.

**Table 4:** Multivariate analysis of clinical swallowing performance trajectories in subacute post-stroke dysphagia patients.

Class	Variables	Reference value	β	SE	Wald χ^2^	*P*	OR	95% CI
C1 vs. C2	Constant term		−4.348	2.822	2.375	0.123		
	Age		0.094	0.045	4.378	0.036	1.098	1.006–1.199
	OHAT		0.180	0.108	2.755	0.097	1.197	0.968–1.481
	Depression: no	Yes	−1.328	0.542	6.000	0.014	0.265	0.092–0.767
	Fatigue: no	Yes	−1.864	0.499	13.947	< 0.001	0.155	0.058–0.412
C1 vs.C3	Constant term		−9.869	3.551	7.724	0.005		
	Age		0.156	0.056	7.859	0.005	1.168	1.048–1.303
	OHAT		0.499	0.127	15.441	< 0.001	1.647	1.284–2.113
	Depression: no	Yes	−2.396	0.691	12.026	0.001	0.091	0.024–0.353
	Fatigue: no	Yes	−3.249	0.737	19.415	< 0.001	0.039	0.009–0.165

C1, mild-rapid improvement group; C2, moderate-steady improvement group; C3, severe-slow improvement group; reference: C1, mild-rapid improvement group.

## 4 Discussion

### 4.1 There are three categories of clinical swallowing performance trajectories in subacute stroke patients with dysphagia

The results of this study showed that clinical swallowing performance improved progressively in patients with post-stroke dysphagia admitted to the rehabilitation department during the subacute phase, as reflected by decreasing SSA scores over time. LCGM identified three distinct trajectory classes of clinical swallowing performance in this patient population: the mild-rapid improvement group, the moderate-steady improvement group, and the severe-slow improvement group, indicating substantial heterogeneity in improvement patterns. The severe-slow improvement group (28%) exhibited gradual improvement in clinical swallowing performance from T1 to T5; however, this improvement was less marked than that of both the moderate-steady improvement group and the mild-rapid improvement group. This pattern may be attributable to severe baseline impairment severity affecting the swallowing neural network—including cortical swallowing areas, corticobulbar tracts, and brainstem central pattern generators (8, 11) such impairment precluded effective compensatory reorganization in perilesional and contralesional regions (24). In addition, standardized rehabilitation stimulation may have been insufficient to elicit functionally relevant neuroplastic changes in patients with severe impairment. Furthermore, the moderate-steady improvement group (41%) accounted for the largest proportion. Although some patients showed improvement in clinical swallowing performance, overall SSA scores remained above the cutoff for dysphagia risk. This may reflect repeated activation of swallowing-related corticobulbar circuits (25). Such activation facilitates neuroplastic mechanisms that enhance synaptic transmission efficiency and optimize functional connectivity within residual neural circuits (26), resulting in partial improvement in clinical swallowing performance. By the end of the 4-week follow-up, however, this group may not yet have reached a plateau. In contrast, the mild-rapid improvement group (31%) showed marked improvement between T1 and T3, possibly due to younger age, better adherence, and higher rehabilitation engagement, which may have enhanced rehabilitation responsivenes (27). However, improvement plateaued from T3 to T5. This plateau may partially reflect the temporal dynamics of neuroplasticity, which reaches maximal activity during the early subacute phase and may subsequently attenuate in capacity for driving further functional gains (11). Additionally, as SSA scores approached the lower detection limit, this plateau may partially reflect a floor effect of the instrument.

### 4.2 Factors influencing clinical swallowing performance trajectories in subacute stroke patients with dysphagia

A systematic review (28) identified age as a predictor of swallowing outcomes in patients with post-stroke dysphagia. The present study further found that advancing age significantly increased the probability of being categorized into the moderate-steady improvement group and the severe-slow improvement group (Table 4). The observed slower improvement in older patients may be explained by mechanisms related to degeneration of the nervous system, which reduces nerve conduction velocity, impairs neural regulation of swallowing, and diminishes neuroplasticity (29). Additionally, age-related declines in swallowing reflex speed and sensitivity may contribute to delayed reflex initiation (7). In addition to these central and reflex mechanisms, physiological changes—including reduced skeletal muscle mass and salivary secretion—may further compromise bolus manipulation and airway protection (8, 30), thereby contributing to slower trajectories of clinical swallowing performance improvement. These results indicate that advanced age may serve as a marker for early risk stratification, helping to identify patients at higher risk who may need more intensive monitoring.

Poor oral health is highly prevalent among patients with dysphagia (31). In this study, each one-point increment in OHAT score was associated with 1.647-fold increased odds of classification into the severe-slow improvement group, compared with the mild-rapid improvement group (OR = 1.647, *P* < 0.001). This finding is consistent with the findings of Peng et al. (32), who identified poor oral health as a risk factor for dysphagia. Similarly, Shiraisi et al. (13) found that improvements in oral health were positively correlated with improvement in clinical swallowing performance. Impaired oral function—such as inflammation and pain—may reduce patient adherence to rehabilitation training. Low adherence could limit the repetitive sensorimotor input required to induce synaptic plasticity within cortico—brainstem pathways and might attenuate rehabilitation responsivenes (27). Poor oral health may also increase the risk of malnutrition (33), which may constrain neural repair capacity. The combined effects of these factors may help explain why poor oral health is more frequently associated with the severe—slow trajectory. Although oral health status showed prognostic relevance for trajectory classification, this observational study cannot establish whether improving oral care modifies these trajectories. Nevertheless, incorporating oral health assessment into swallowing care protocols may facilitate individualized care planning by addressing modifiable barriers.

The results of this study showed that, compared with subacute post-stroke dysphagia patients without fatigue, those with fatigue are more likely to be classified into the moderate-steady improvement group and the severe-slow improvement group, suggesting that fatigue is an unfavorable factor for improvement in clinical swallowing performance in this population. Previous studies (34, 35) have found that dysphagia increases the risk of post-stroke fatigue, while fatigue in turn exerts a negative impact on clinical swallowing performance. Post-stroke fatigue and dysphagia may share common neural mechanisms involving the primary motor cortex (7, 36); dysfunction within this shared network may attenuate the expression of experience-dependent neuroplasticity (7). Moreover, fatigue reduces patients' willingness and ability to engage actively in rehabilitation, which limits active participation in swallowing exercises and thereby impedes improvement in clinical swallowing performance. These findings suggest that screening for fatigue may help identify patients who may require consideration of training intensity and timing adjustments, thereby supporting individualized rehabilitation planning.

Depression is a prevalent neuropsychiatric complication among stroke survivors and is associated with unfavorable functional outcomes (37). This study found that depression was an independent predictor of unfavorable SSA score trajectories; compared with patients without depressive symptoms, those with concomitant depression were more likely to be categorized into the moderate-steady improvement group and the severe-slow improvement group (Table 4). Patients with depression often take antidepressants or sedatives, which inhibit salivary secretion and reduce oral lubrication, thereby increasing frictional resistance during swallowing and elevating the risk of aspiration (38). Depression can reduce appetite, trigger fear of eating, and lead to social withdrawal, all of which may undermine nutritional status and swallowing practice. Crucially, diminished rehabilitation adherence attenuates rehabilitation responsiveness (27), ultimately hindering gains in clinical swallowing performance. These findings suggest that screening for depression may support individualized care planning regarding psychological support needs.

### 4.3 Limitations

This study identified heterogeneous clinical swallowing performance trajectories in subacute stroke patients with dysphagia admitted to rehabilitation departments, and examined their determinants. However, several limitations should be noted. First, no longitudinal instrumental swallowing assessments were conducted. The SSA relies on observable signs such as coughing and voice changes; FEES, by contrast, permits direct visualization of physiological swallowing parameters such as pharyngeal residue, penetration, or silent aspiration. Because this study used FEES only at baseline to confirm dysphagia and relied exclusively on the SSA for all follow-up assessments, the identified trajectories should be interpreted as patterns of change in clinical swallowing performance rather than as definitive evidence of physiological swallowing recovery. Patients in favorable trajectories may still have experienced unrecognized silent aspiration or persistent pharyngeal residue. Second, although a rehabilitation protocol was provided in this study, we did not systematically record the exact duration of orofacial muscle training and postural swallowing maneuvers, nor did we quantify inter-therapist variability or session-by-session adherence. Consequently, it is difficult to ascertain whether the differences among trajectories reflect intrinsic patient characteristics or variations in treatment implementation. Third, this study did not account for lesion location—particularly brainstem involvement. Given that brainstem stroke is a well-established predictor of swallowing outcomes, this omission may have introduced unmeasured confounding, potentially biasing the association between the identified trajectories and their determinants. Fourth, this study focused primarily on changes in SSA scores and did not capture clinically meaningful outcomes such as the Functional Oral Intake Scale (FOIS) scores, feeding tube removal, aspiration pneumonia, nutritional recovery, or hospital length of stay, somewhat limiting the clinical interpretation of these trajectories. Finally, the single-center design and small sample size limit statistical power and generalizability, and may obscure small effects in subgroup analyses.

## 5 Conclusion

Using the SSA, this study identified three distinct trajectories of clinical swallowing performance in subacute stroke patients with dysphagia admitted to rehabilitation units: mild-rapid improvement, moderate-steady improvement, and severe-slow improvement. These trajectories indicate marked heterogeneity in how clinical swallowing performance changed over time. Age, oral health status, fatigue, and depression predicted trajectory membership, with fatigue and depression linked to poorer clinical swallowing performance. These preliminary findings suggest that trajectory classification may assist in early risk stratification at admission and may support individualized care planning. However, because this study employed an observational design, it does not demonstrate that trajectory-based management improves patient outcomes. Future interventional studies, such as randomized controlled trials, are needed to evaluate whether trajectory classification confers clinical benefit.

## Data Availability

The raw data supporting the conclusions of this article will be made available by the corresponding author, without undue reservation.
